# P-815. Clinical Impact of BioFire® Blood Culture Identification 2 Implementation in Patients with Staphylococcal Bacteremia

**DOI:** 10.1093/ofid/ofae631.1007

**Published:** 2025-01-29

**Authors:** Colin Duell, Tyler Ackley, Kristin E Linder, Joseph L Kuti, Alivia Castle, Rosanna Li

**Affiliations:** Hartford Hospital, Glens Falls, New York; Hartford Hospital, Glens Falls, New York; Hartford HealthCare, Hartford, Connecticut; Hartford Hospital, Glens Falls, New York; The University of Saint Joseph, Hartford, Connecticut; Maimonides Medical Center, New York, New York

## Abstract

**Background:**

The BioFire® Blood Culture Identification 2 (BCID2) PCR panel aids in rapid identification of blood pathogens, including coagulase-negative Staphylococci (CoNS) and methicillin susceptible *Staphylococcus aureus* (MSSA). BCID2 was implemented at all Hartford HealthCare hospitals in September 2022. The objective of this study was to evaluate how BCID2 impacted vancomycin use, clinical outcomes, and pharmacist intervention.

**Methods:**

This was a multi-center, retrospective study of adult patients admitted to Hartford HealthCare hospitals with MSSA or CoNS positive blood cultures. The primary endpoint, days of vancomycin therapy, was compared between the pre- (3/7/22 – 9/7/22) and post-BCID2 periods (10/7/22 – 4/7/23). Secondary endpoints included duration of MSSA positive blood cultures, intensive care unit (ICU) length of stay, hospital mortality, and pharmacist interventions accepted.

**Results:**

Of the 617 patients included in the primary analysis, MSSA bacteremia accounted for 37.0% (110/297) and 31.6% (101/320) of the patients in the pre- and post-BCID2 group, respectively (p=0.178). The median duration of vancomycin was 1.3 and 0.3 days in the pre- and post-BCID2 group, respectively (absolute difference, 1.0 days; p< 0.001). The duration of MSSA BSI was 3.1 and 2.4 days in the pre- and post-BCID2 group, respectively (absolute difference, 0.7 days; p=0.096). Hospital mortality was not different between periods [7.1% (21/297) vs 10.6% (34/320), p=0.16]. In patients with MSSA bloodstream infection (BSI), ICU LOS was 4.4 days shorter in the post-BCID2 period (7.8 vs 3.4 days, p< 0.001). Pharmacists successfully intervened to reduce vancomycin duration in 7.1% (21/297) and 16.3% (52/320) of patients in the pre- and post-BCID2 group, respectively (p< 0.001).

**Conclusion:**

BCID2 implementation shortened the duration of vancomycin in patients with MSSA or CoNS bacteremia and facilitated pharmacist intervention to stop vancomycin in non-pathogenic CoNS positive blood cultures or switch to MSSA-targeted therapy in MSSA BSI. These data support BCID2 as a critical tool to improve appropriate antibiotic selection and shorten the time to safer and more effective treatment for patients with CoNS and MSSA bacteremia.

**Disclosures:**

**Joseph L. Kuti, PharmD**, Abbvie: Advisor/Consultant|bioMerieux: Grant/Research Support|Merck: Grant/Research Support|Pfizer: Grant/Research Support|Shionogi Inc: Advisor/Consultant|Shionogi Inc: Grant/Research Support|Shionogi Inc: Honoraria|Venatorx: Grant/Research Support

